# „Wider die Natur“ – Zur sozialpsychologischen Dimension des Bündnisses von Verschwörungsdenken und Spiritualität in den Corona-Protesten. Eine Fallanalyse

**DOI:** 10.1007/s41682-023-00150-7

**Published:** 2023-03-22

**Authors:** Florian Knasmüller, Gero Menzel, Tobias Reuss, Markus Brunner, Ayline Heller

**Affiliations:** 1 Fakultät für Psychologie, Sigmund Freud PrivatUniversität, Wien, Österreich; 2 Fakultät für Psychotherapiewissenschaft, Sigmund Freud PrivatUniversität, Wien, Österreich; 3 Fachbereich Katholische Theologie, Goethe-Universität, Frankfurt, Deutschland; 4 International Psychoanalytic University Berlin, Berlin, Deutschland; 5 Klinik und Poliklinik für Psychosomatische Medizin und Psychotherapie, Universitätsmedizin der Johannes Gutenberg-Universität Mainz, Mainz, Deutschland

**Keywords:** Conspirituality, Corona-Proteste, Tiefenhermeneutik, Schiefheilung, Verschwörungsdenken, Esoterik, Conspirituality, COVID-19 protests, Depth-hermeneutics, Crooked cure, Conspiracy beliefs, Esotericism

## Abstract

Seit dem Aufkommen lautstarker und öffentlichkeitswirksamer Proteste gegen die Corona-Politik wird immer öfter das Konzept der *conspirituality *bemüht, um den ideologischen Kitt des heterogenen Protestmilieus theoretisch zu bestimmen. Im vorliegenden Artikel wird zum einen nachgezeichnet, wie sich verschwörungs- und esoterisch-spirituelles Denken in der conspirituality miteinander verzahnt und wie diese Vorstellungs- und Gefühlswelten von okkulten Milieus ausgehend diffundierten und allmählich popularisiert wurden. Zum anderen wird anhand einer tiefenhermeneutischen Einzelfallanalyse eines biografisch-narrativen Interviews mit einer Protestteilnehmerin gezeigt, dass die Ideologiefragmente auf idiosynkratische Weise angeeignet und in die eigenen Deutungssysteme eingeflochten werden. Durch diese Herangehensweise gerät weiterhin in den Blick, welche Unsicherheiten durch die Pandemie und die politischen Versuche dieser Herr zu werden ausgelöst wurden und wie diese zu lebensgeschichtlichen Erfahrungs- und Beziehungsmustern der Interviewten stehen. Vor diesem Hintergrund, so das Fazit der Studie, dient die conspirituality als *Schiefheilungsschablone*, die es erlaubt gesellschaftlich (mit-)produzierte, innere Konfliktlagen zu bearbeiten und abzufedern, indem unerträgliche Affekte, Ambivalenzen und Ängste, aber auch unerfüllte Wünsche nach Harmonie und Geborgenheit projektiv entweder in die Natur gelegt oder bösartigen Verschwörer:innen zugeschrieben werden.

## Abstract (en)

In the light of increasingly loud and highly visible public protests against protective measures and policies against COVID-19, the concept of *conspirituality* has recently gained a lot of attention. It is used to theoretically grasp the ideological glue of the heterogeneous milieu of protesters. The aim of this article is twofold. First, we show how, in conspirituality, elements of conspiracy beliefs are intertwined with esoteric-spiritual ideas. Going back to occultic milieus, these worldviews are then diffused and slowly popularized. Second, using depth-hermeneutic analyses of a biographical interview with a protest participant, we show that fragments of ideology are ingested in an idiosyncratic manner and interlaced with existing subjective interpretive patterns. This will further reveal the fundamental insecurities caused by the pandemic itself and by the political attempts to deal with its effects. Against this background, we conclude that conspirituality serves as a pattern of ‘crooked cure’, mitigating inner conflicts (co-)produced by society. This is achieved by protectively ascribing unbearable affects, ambivalences, and anxieties, but also unfulfilled desires of harmony, security, and comfort either to nature or to malignant conspirators.

## Einleitung

Als sich der Covid-19 Erreger ab Februar 2020 auch in Europa rasant zu verbreiten begann, regte sich zunächst kaum öffentlich wahrnehmbarer zivilgesellschaftlicher Widerstand gegen die Maßnahmen, die von Staats wegen zur Eindämmung des Virus verhängt wurden. Erst als die *erste Welle* der Pandemie abflachte, übersetzte sich der Unmut, der sich in digitalen Räumen zusammen gebraut hatte, auch in erste Protestaktionen auf der Straße. Heute lässt sich konstatieren, dass die sog. *Corona-Proteste *mehr sind als das sporadische Aufflackern sozialer Unzufriedenheit im öffentlichen Raum: Die Proteste haben sich vielerorts zu einem langlebigen Phänomen entwickelt, das einerseits die öffentliche Debatte um die Angemessenheit der Pandemiemaßnahmen mitgeprägt hat und andererseits selbst zum Gegenstand von kontroversen Auseinandersetzungen über die Rechtmäßigkeit der Protestformen, Anliegen und die politische Einordnung der Protestierenden wurde. Die eigentümliche Zusammensetzung der Kundgebungen rief im öffentlichen Diskurs bereits früh Irritationen hervor: Dort fanden sich von rechten bis extrem-rechten Akteur:innen über die vermeintliche gesellschaftliche Mitte bis hin zu alternativ-esoterischen Milieus Gruppen ein, die auf den ersten Blick kaum eine ideologische Schnittmenge aufzuweisen schienen. Symbolisch spiegelte sich diese *Querfront *bspw. im gleichzeitigen und unwidersprochenen Nebeneinander von Reichskriegsfahnen, Q‑Anon-Symbolen, Regenbogenfahnen, religiösen Symbolen und Markern der Esoterik- bzw. ‚Hippie‘-Szene.

Erste Einschätzungen über den ideologischen Kitt der Protestszene haben sich mittlerweile zu einem eindeutigeren Bild verdichtet: Die Protestierenden eint die Oppositionshaltung gegenüber dem ‚Mainstream‘, einer Kategorie unter der alles subsumiert wird, was mit denen ‚da oben‘ assoziiert wird, einer vermeintlichen Elite und den, so die Imagination, von dieser abhängigen und ihr deshalb hörigen etablierten Massenmedien und wissenschaftlichen Institutionen. Gegen diese wird ein „Gegenwissen“ in Anschlag gebracht, dessen Legitimation aus dem „Bauchgefühl“, durch eigene Recherchen in „alternativen“ Medien und aus den Einschätzungen szeneinterner Expert:innen abgeleitet wird, was die Entstehung einer „Misstrauensgemeinschaft“ begünstigt (Pantenburg et al. 2021, S. 29 ff.). Zusammengehalten wird diese Gemeinschaft hingegen nicht alleine durch eine gefühlte Wahrheit über die Pandemie und Maßnahmen zum Infektionsschutz, sondern ganz zentral durch verschwörungsideologische und esoterisch-spirituelle Weltsichten: So konnten quantitative Untersuchungen zeigen, dass Verschwörungsdenken in den Protestmilieus weite Verbreitung findet und darüber hinaus auch esoterische Einstellungsmuster in den geteilten Weltbildern zentral verankert sind (Brunner et al. 2021; Nachtwey et al. 2020). Den Befragten erscheint der politische Umgang mit der Pandemie in Österreich, Deutschland und der Schweiz fast einhellig als Ausdruck gezielter Instrumentalisierungsversuche zur Überwachung und Kontrolle der Bevölkerung, wobei Wissenschaftler:innen und Journalist:innen vorgeworfen wird, zu diesem Zweck Angst und Desinformation zu verbreiten. Diesen imaginierten Manipulationsversuchen und Verschwörungen halten die Demonstrierenden eine abstrakt gehaltene Naturverbundenheit, die natürlichen Selbstheilungskräfte des Menschen sowie spirituelles und ganzheitliches Denken entgegen – Denkmuster, die eine klare Einteilung in ‚gut‘ und ‚böse‘ und somit ein Angebot zur Komplexitätsreduktion liefern (vgl. Butter 2018). In der Literatur wird ob dieser Befunde diskutiert, wie sich in der Protestszene esoterisch-spirituelle und verschwörungsideologische Einstellungsmuster zueinander verhalten bzw. inwiefern beide miteinander verzahnt sind (vgl. Schäfer und Frei 2021; Schließler et al. 2020). Innerhalb der Religionswissenschaften wird die Verschränkung besagter Ideologien unter dem Konzept der *conspirituality *verhandelt (Asprem und Dyrendal 2015; Ward und Voas 2011). Im Anschluss an diese Debatten wollen wir im Folgenden *erstens *das Verhältnis von Verschwörungsdenken und Esoterik bzw. Spiritualität innerhalb der conspirituality herausarbeiten und anhand einer historischen Betrachtung ergründen, inwieweit es sich bei diesen Verzahnungen um neue Formen von Spiritualität handelt, *zweitens *anhand einer tiefenhermeneutischen Falldarstellung rekonstruieren, auf welche spezifische Art und Weise sich Elemente dieser Denkformen in den Erzählungen und Deutungsmustern der Protestierenden manifestieren *können*, um *drittens *unter Berücksichtigung psychoanalytisch-sozialpsychologischer Konzepte der psychischen Funktion von conspirituality nachzugehen.

## Begriff und Bedeutung

Die folgenden Ausführungen schließen wesentlich an Schließler et al. (2020) an, deren Überlegungen wir hier weiterführen wollen. Wie diese greifen wir das Konzept der conspirituality auf, welches auf Ward und Voas (2011) zurückgeht, um Dynamiken der Corona-Proteste zu konzeptualisieren. Jene begreifen conspirituality, ein Portmanteau-Wort, welches *conspiracy* und *spirituality* zusammenfügt, als neuartige Verbindung von rechtsgerichtetem, weißem und stereotyp-männlichem Verschwörungsdenken und (linksgerichteter) stereotyp-weiblicher New-Age-Spiritualität (S. 105 ff.). Im Anschluss daran verweisen Asprem und Dyrendal (2015) in einer kritischen Reformulierung des Konzepts auf die historische Kontinuität dessen, was unter den Begriff der conspirituality subsumiert wird: Ihre zentrale These ist, dass Verschwörungsdenken immer schon immanenter Teil dessen war, was unter *spirituality* gefasst wird.

Zuerst ist zu konstatieren, dass der inhaltliche Zusammenhang des Phänomens um die drei Paradigmen kreist, die sowohl esoterischem als auch verschwörungsideologischem Denken grundlegend sind: „Nothing happens by accident“, „nothing is as it seems“ und „everything is connected“ (Barkun 2013, S. 3 f.). Sie bilden die Matrix, entlang derer sich die Inhalte aus Esoterik mit Elementen des Verschwörungsdenkens zusammenfügen und als spezifische Verschaltung von Ideologemen erscheinen. Ergebnis ist die Entstehung eines holistischen Weltbildes, in das die verschiedenen Versatzstücke von Verschwörungsideologien ebenso integriert werden können wie Momente esoterisch-spiritueller Narrative, und für das zudem eine Gleichzeitigkeit von Rationalismus und Irrationalismus kennzeichnend ist. Während die Aspekte des Verschwörungsdenkens versuchen, ihre Umwelt mit einer überformten Rationalität zu erklären, greifen die esoterisch-spirituellen Aspekte auf Momente von Leiblichkeit oder der Wahrheit eigener Gefühle und Affekte beim Erkennen äußerer Realität zurück. Gerade letzteres kehrt dabei die „mystische, religiöse oder spirituelle Dimension“ (Schink 2020, S. 407) hervor, die kennzeichnend für das Phänomen der conspirituality ist.

Ein zentrales Moment der conspirituality ist dabei die psychodynamische Dimension der Projektion, die sich sowohl im esoterisch-spirituellen Denken selbst (Asprem und Dyrendal 2015, S. 376) als auch im Verschwörungsdenken bereits ankündigt (Moscovici 1987): Negative, unerwünschte Selbstanteile werden beim anderen im Außen verortet und dort bekämpft bzw. kontrolliert. So können einerseits Erfahrungen eigener Einschränkungen und eigenen Verzichts projektiv an ‚den anderen‘ bearbeitet werden (ebd., S. 160 f.), andererseits können auch eigene Marginalisierungserfahrungen durch die Konstruktion eines epistemischen Gegennarratives, welches das eigene Wissen als richtiges gegenüber einem ‚falschen‘ und ‚verklärten‘ Wissen der Mehrheitsgesellschaft setzt, projektiv bearbeitet werden (Asprem und Dyrendal 2015, S. 372; Dyrendal 2015; Dyrendal et al. 2019, S. 28). Dadurch ergibt sich eine klare Trennung zwischen dem ‚bösen Außen‘ und dem ‚guten Selbst‘, verbunden mit einer Aufwertung der eigenen Position. Hinter der Projektion, die als Abwehrbewegung im psychoanalytischen Sinn verstanden werden kann, kann dabei u. a. der Wunsch stehen, die Unkontrollierbarkeit und Undurchsichtigkeit der modernen Gesellschaft zurückzuweisen und stattdessen eine als kontrollierbar erlebte Welt oder einen versöhnten „(Ur‑)Zustand“ mit der Natur zu erleben oder anzustreben. Im Verschwörungsdenken sind es v. a. die vermeintlich mächtigen, kleinen Gruppierungen, die für Probleme und Konflikte in der Welt verantwortlich gemacht werden, während es in spirituellen Denktraditionen v. a. ein allgemeines Prinzip ist, das bspw. auf die moderne Gesellschaft projiziert wird und dann in der Projektionsbewegung eine „böse deformierende Gesellschaft“ einer „guten Natur“ (Schließler et al. 2020, S. 293) gegenübersteht. In der conspirituality können diese beiden Projektionsflächen zusammenfallen.

Neben der Projektion steht deshalb eine identifizierende Bewegung, in der sich die Individuen mit Mächten gemein machen möchten, die als Gegenpol zu einer entfremdeten, in Teilen unverständlichen Gesellschaft, hypostasiert werden (Adorno 2003). Hier wird die Natur zum positiven Identifikationsobjekt, mit dem sich das Individuum identifizieren kann und die Rat, Trost und Schutz bietet, so lange man fähig ist, ihr Wesen zu verstehen und ihre Erscheinungen zu deuten. In der conspirituality ist demnach der oben beschriebene Wunsch nach einem vermeintlich spannungsfreien, symbiotischen Zustand mit der Natur auch immer mit einer Suche nach Sinn und Wahrheit verbunden. Sie kann als Versuch verstanden werden, sowohl Angst und eigene Überforderung zu binden als auch narzisstische Kränkungen, die den Individuen durch eine der unmittelbaren Kontrolle entzogenen Welt zugefügt werden, zu kompensieren (Schließler et al. 2020, S. 293).

Diese identifizierende Bewegung findet sich im Konzept der conspirituality im Begriff der *spirituality* wieder. Spiritualität wird als Gegenbegriff zu (institutionalisierter) Religion verstanden (Barker 2004; Knoblauch 2010, S. 162; Partridge 2014, S. 113). Dadurch rücken die ‚gelebte Religion‘ und die subjektiv-psychologische Dimension in den Fokus (Roof 2003, S. 142 ff.). In diesem Rahmen lassen sich subjektive Praktiken und Narrative des *(re-)enchantment* bzw. der Sakralisierung betrachten (Ammerman 2020; Gould 2005; Palmisano und Pannofino 2021), in denen alltägliche Praktiken und Erfahrungen mit Sinn erfüllt werden. Außerdem ermöglicht der Begriff der Spiritualität eine sozialpsychologische Perspektive auf Sinngebungsdynamiken, da er die subjektive Verhältnisbestimmung zum Sakralen im Gegensatz zu einer institutionell vermittelten in den Mittelpunkt rückt (Palmisano und Pannofino 2021, S. 24).

Um die genealogische Argumentation von Dyrendal und Asprem aus der historischen Bewegung des Begriffs zu entfalten und um eine Einbettung des untersuchten Einzelfalles zu ermöglichen, soll im Folgenden die historische Bewegung von *untergründiger *über *alternative *zu einer *populären *Spiritualität (Knoblauch 2010) und ihre jeweilige Verbindung zum Verschwörungsdenken nachgezeichnet werden.

## Zur Genealogie der Conspirituality

Obwohl die Verbindung von Verschwörungsdenken und Spiritualität gerade im Kontext der sog. Corona-Proteste augenscheinlich wird, lässt sich das Phänomen nicht nur über seinen Bezug zum 21. Jahrhundert begreifen, sondern reicht in seiner Genese bis zu den Anfängen der Epoche der Aufklärung zurück. Esoterische Strömungen entwickelten sich hier mit und neben aufklärerischem Denken. Ebenso nahmen Denker:innen der Aufklärung immer wieder impliziten Bezug auf hermetische und theosophische Denkansätze oder stellten mit der Orientierung an Gedanken in christlich-platonischer Tradition Bezüge zu ‚altem Wissen‘ her (Neugebauer-Wölk 2013; Neugebauer-Wölk und Meumann 2013, S. 18 ff.).

Dabei ist den esoterischen Strömungen in ihrer Abgrenzung zur Bewegung der Aufklärung gemein, dass sie Bezug nehmen auf vermeintlich ausgegrenztes, untergründiges Wissen. Sie treten in Opposition zur auf Vernunft und Rationalität fußenden Denktradition und imaginieren sich im selben Zug als Bewahrer ‚alten Wissens‘:

„The dynamics of rejected knowledge that thus came into play has allowed self-defining occultist to understand themselves as standing in opposition to an Establishment working against the true, liberating wisdom of the ancients“ (Asprem und Dyrendal 2015, S. 374).

Folgt man der Logik einer Verteidigung marginalisierten Wissens, lassen sich hier die Anknüpfungsstellen zum Verschwörungsdenken finden, die der Traditionslinie der *occultists* bis heute innewohnen und das Phänomen der conspirituality vorbereiten. Mit dem ausgehenden 18. Jahrhundert etabliert sich in Europa und den USA ein sog. *occultic milieu*, das von Anfang an Schlüsselgedanken des Verschwörungsdenkens übernimmt und in die eigenen Vorstellungswelten integriert und gleichzeitig selbst Ort der Verbreitung und Anverwandlung bestehender verschwörungsideologischer Elemente ist. Inhaltlich lässt sich von diesem Milieu ausgehend eine Kontinuität herausarbeiten, die das occultic milieu des 18. Jahrhunderts mit dem sog. *cultic milieu* des 20. Jahrhunderts verbindet. Jenes findet in der Popularisierungstendenz esoterischen Wissens und esoterischer Praktiken der New Age-Bewegung^1^ seinen vorläufigen Höhepunkt (Knoblauch 2009, S. 100 ff.).

Auch wenn in diesem weiten Feld sehr verschiedene Theorien und Vorstellungen kursieren und esoterische und mystische Praktiken eine individuelle und vereinzelnde Tendenz haben, geht aus diesem mystisch-spirituellen Milieu ein negativ-vergemeinschaftender Effekt aus der Abgrenzung hervor. Er bleibt allerding temporär, weshalb die Gruppierungen fluktuieren. So lässt sich das dort kursierende Wissen als „heterodox or deviant items in relation to the dominant cultural orthodoxies“ (Campbell 1972) verstehen – es markiert und verweist auf die Verbindungslinien, die zwischen dem *cultic* und occultic milieu gezogen werden können.

Während das *oc/cultic milieu* eine Subkultur bildet und als *untergründige Spiritualität *beschrieben wird, wird die damit verbundene New-Age-Spiritualität unter dem Gesichtspunkt einer Verbreitung und Popularisierung dieser Spiritualität besprochen (Knoblauch 2010; Partridge 2014). Nach Knoblauch durchläuft die New-Age-Bewegung alle diese Stufen: Ihr Beginn findet sich in einer „oppositionellen Gegenkultur“ (Knoblauch 2010, S. 159), darauf verbreitet sich das New Age-Denken und findet zunehmend soziale Anerkennung, es etabliert sich daran anschließend durch die strukturelle Vermeidung von hierarchischen Organisationsformen in Opposition zu dominanten religiösen Strukturen als *alternative Spiritualität*. Leitgedanke ist hier, dass sich eine individuelle Veränderung zu einer gesellschaftlichen Veränderung steigern soll (ebd., S. 158 ff.). Unter zeithistorischer Perspektive beschreibt Knoblauch in den 1980er bis zu den 2000er Jahren eine Expansion des Milieus alternativer Spiritualität, indem „esoterische Kultur im Umfeld des New Age […] in den verschiedensten Medien, in populären Fernsehsendungen, Filmen, Büchern und Buchserien einem breiteren Publikum zugänglich gemacht“ (Knoblauch 2009, S. 103 f.) wird, bis sich eine Eingrenzung auf ein Milieu nicht mehr aufrechterhalten lässt. Kriterium der Unterscheidung ist dabei, dass sich die „Praktiken, die einst unter dem New Age zusammengefasst wurden“ (Knoblauch 2010, S. 166), nicht mehr auf eine scharf vom kirchlichen Milieu getrennte Gruppe begrenzen lassen, sondern in populären Kultur- und Mainstream-Diskursen zirkulieren und deshalb bisweilen unter den Begriff der *populären Spiritualität* eingeordnet werden müssen (Hanegraaff 1996, S. 1 ff.; Knoblauch 2010, S. 163 ff.). Der Bereich der alternativen Spiritualität geht allerdings nicht vollständig in der populären Spiritualität auf, vielmehr stehen Popularisierung und Alternativität in einem dialektischen Verhältnis und Akteur:innen der Alternativität und der Popularisierung sind um gegenseitige Abgrenzung bemüht. Weiterhin befinden sich die Praktiken popularisierter Spiritualität in einem gesellschaftlichen Legitimierungsdiskurs mit Politik und Medizin, der um die Frage von wissenschaftlicher und juristischer Anerkennung auf der einen und Abgrenzung auf der anderen Seite kreist. Diese populäre Spiritualität begründet sich auf heterodoxen und devianten Gegenständen, die sie gleichzeitig als bessere bzw. heilsbringende Alternative etablieren möchte. Sie ist verbreitet, aber institutionell kaum anerkannt (Knoblauch 2009, S. 104).

Im Zuge dieser Verbreitungs- und Popularisierungsbewegung diffundieren Vorstellungen und Inhalte des oc/cultic milieu als populäre Spiritualität immer mehr in popkulturelle Erzeugnisse und werden in diesem Kontext Teil kulturindustrieller Produktion. Die Kulturalisierung zentraler Momente von Spiritualitätsvorstellungen lässt sich dabei, Asprem und Dyrendal folgend, als Phänomen der „Occulture“ (Asprem und Dyrendal 2015, S. 368; Partridge 2014) beschreiben und bildet die Verlängerung der Traditionslinie des mit und gegen die Aufklärung entstehenden occultic milieus inklusive der damit verbundenen Gedankenwelt. Die Affinität, die diesen Inhalt im Hinblick auf ihre Synthese und Verbindung mit verschwörungsideologischen Denkmustern anhaftet, verbreitet sich mit (Partridge 2014, S. 116 f.) und getrennt von ihnen als *conspiracy culture* (Barkun 2013).

Conspirituality kann als eine dynamische Verbindung von Spiritualität und Verschwörungsdenken angesehen werden, das besonders im Rahmen der politischen Religion bzw. Spiritualität von Bedeutung ist (Asprem und Dyrendal 2015, S. 370; Aupers und Harambam 2019; Dyrendal 2015, S. 286; Ward und Voas 2011). Auf theoretischer Ebene konnte von uns gezeigt werden, dass die Basis für das Konzept der conspirituality in seinem strukturellen Potenzial zur Verbindung von Spiritualität und Verschwörungsdenken sowie in den ihnen immanenten Vorstellungen von Welt und deren Interpretation begründet liegt. Darüber hinaus wurde auch auf psychologischer Ebene deutlich, dass beide Formen ähnliche Momente und Bedürfnisse der Individuen ansprechen. Historisch konnten wir nachzeichnen, dass die Verbindung von Spiritualität und Verschwörungsdenken nicht nur eine theoretische ist, sondern sich als Traditionslinie von der Aufklärung bis in das 21. Jahrhundert hinein vollzieht. Die Verbreitung von conspirituality im Kontext der sog. Corona-Proteste knüpft sowohl unter einem theoretischen als auch unter einem historischen Gesichtspunkt an die aufgezeigten Bewegungen und Dynamiken an. Im Kontext der nachfolgenden Fallanalyse soll deshalb conspirituality als zentrale Kategorie den Rahmen bilden, in den die virulenten Dynamiken eingeordnet werden können.

## Methodisches Vorgehen

Beim vorgestellten Material handelt es sich um ein biografisch-narratives Interview (Schütze 1983) mit einer deutschen Staatsbürgerin, die sich an Corona-Protesten in Deutschland beteiligt hat. Das Interview wurde mit der *Tiefenhermeneutischen Kulturanalyse* nach Alfred Lorenzer (1986) ausgewertet. Dieses psychoanalytisch informierte Auswertungsverfahren operiert auf zwei Ebenen: Zum einen widmet es sich der Rekonstruktion des *manifesten Gehalts* des Interviewmaterials. Darunter wird verstanden, wie die Interviewte sich selbst, ihre Lebensgeschichte und ihre Position in der Welt begreift, einordnet und reflektiert. Zum anderen geht die Tiefenhermeneutik davon aus, dass sich im Material untergründig eine *latente Bedeutungsebene* Ausdruck verschafft, die in einem Spannungsverhältnis zum manifesten Sinn steht. Neben dem, was die Interviewten intentional über sich preisgeben und logisch verstanden werden kann, lassen sich durch* szenisches Verstehen* auch weniger bewusste Motive, Wünsche und Affekte freilegen, die das Material durchdringen. Diese bleiben auf der manifesten Ebene unbenannt, weil sie gesellschaftlich verpönt sind und/oder der eigenen Selbstwahrnehmung widersprechen. Folglich ist die Tiefenhermeneutik mit ihrem Fokus auf der sozialpsychologisch bedeutsamen Spannung zwischen manifester Meinungsartikulation und deren latent-affektivem und emotionalem Gehalt für die Analyse und Auswertung emotionalisierter politisierter Diskurse des Materials sowie deren psychischer Funktion vor dem Hintergrund biografisch erworbener Konfliktlagen besonders gut geeignet.

Als Erkenntnisinstrument dienen der Tiefenhermeneutik Interpretationsgruppen, die sich dem Material zunächst assoziativ nähern, um dann die daraus resultierenden *Gefühlsantworten, *Irritationen, Affekte oder Assoziationen, auf die manifeste Sinnebene zu beziehen – in iterativen Pendelbewegungen zwischen diesen Ebenen kristallisiert sich nach und nach eine stimmige Interpretation des Falles heraus (König 2019). Die Interpretationsgruppen dienen gleichzeitig als Korrektiv: Mit der Aufforderung, Assoziationen und Interpretationsansätze stets ans Material zu binden, helfen sie dabei, subjektive, idiosynkratische Anteile als solche aufzudecken. Die Gefühlsreaktionen werden also keineswegs als Wahrheit über den Fall missverstanden, sondern methodisch kontrolliert als Richtschnur genutzt, die auf latente Dimensionen hinweisen. In unserem Fall wurde das Material zunächst innerhalb der Forschungsgruppe (den Autor:innen) vorstrukturiert und über das szenische Verstehen wurden erste Interpretationsansätze verfolgt. Danach wurde mithilfe von externen Interpretationsgruppen weitere, bis dahin vernachlässigte Lesarten im Material aufgedeckt, um so eine Interpretation des latenten Gehaltes herauszuarbeiten. Die finale Deutung und Verschriftlichung lag wiederum in den Händen der Forschungsgruppe, die die Ergebnisse der Interpretationsgruppe schließlich auf die Forschungsfrage bezog und in den aktuellen Forschungsstand theoretisch einbettete.

Mit dieser *Einzelfallanalyse *(vgl. Hering und Schmidt 2014) beanspruchen wir freilich nicht das Protestmilieu als solches hinlänglich analysieren oder gar verallgemeinerbare Aussagen über die Rolle der conspirituality in diesem treffen zu können. Der Fokus auf den ausgewählten Fall erlaubt uns jedoch einen genaueren Blick darauf, 1) welche inneren (Konflikt‑)Dynamiken durch die Krise und die politischen Regulationsversuche angestoßen werden, 2) wie das Krisenerleben und dessen affektive Niederschläge durch biografische Erfahrungsmuster mitgeprägt wird und nicht zuletzt wie 3) Amalgamierungen von Verschwörungsdenken und Esoterik bzw. Spiritualität zur affektiven Bearbeitung dieser Konfliktlagen angeeignet werden sowie in welcher spezifischen Gestalt diese Vermengung auftreten können. An dem ausgewählten Fall, so sind wir überzeugt, lassen sich bisher unberücksichtigte Facetten der Rolle von conspirituality in der Corona-Protest-Szene herausarbeiten.

Im Folgenden werden wir den latenten Gehalt des Textes, wie er sich auf Basis der Interpretationssitzungen darstellte, in stark verdichteter Form herleiten. Dafür werden wir einzelne Interviewszenen miteinander in Verbindung bringen, um durch deren Konstellierung die im Sinne des szenischen Verstehens entwickelten Interpretationen zu plausibilisieren. Abschließend werden die getätigten Interpretationen vor dem Hintergrund der vorab dargestellten Theorien diskutiert.

## Falldarstellung

Bei dem im Folgenden vorgestellten Fall handelt es sich um eine in etwa 45-jährige Frau, der wir das Pseudonym *Cornelia *gegeben haben. Die Interviewte lebt in einer kleineren Stadt in der ehemaligen DDR auf einem sanierten Bauernhof und arbeitet als Selbstständige im Bereich körpernaher Dienstleistungen. Cornelia wurde über eine quantitative Befragung rekrutiert, in der es Teilnehmer:innen freigestellt wurde, bei Bereitschaft ein qualitatives Interview zu geben, ihre E‑Mail-Adresse zu hinterlassen; Cornelia wurde per Zufallsprinzip ausgewählt. Das Interview wurde im Mai 2021 online durchgeführt, transkribiert und sorgfältig pseudonymisiert.

### Vogelfreie Kindheitserfahrungen

In der DDR geboren worden zu sein, setzt Cornelia zentral, und der Zusammenbruch der DDR, zu dessen Zeitpunkt sie etwas älter als 10 Jahre gewesen sei, markiert nicht nur einen Knotenpunkt in ihrer und der Lebensgeschichte ihrer Eltern, sondern auch in ihrer Erzählung. Von ihrer Kindheit zeichnet Cornelia ein idyllisches Bild, das von aus heutiger Sicht kaum noch auszumalenden Freiheitsgraden geprägt gewesen sei:

wenn ich das vergleiche wie’s jetzt abläuft (.) wir zum Beispiel sind von der Schule nachhause gekommen […] dann hat man seinen Ranzen in die Ecke geschmissen, hat seine Tobesachen angezogen, sich mit seinen Freunden draußen getroffen und war zum Abendbrot (.) zurück (.) und wir haben im Wald getobt (.) früher wurde man ja auch nicht beaufsichtigt (.) wir sind als (.) sag ich mal als Horde Kinder spielen gewesen^2^

Nach der Schule habe man sich mitsamt dem Ranzen auch dem Zugriff durch einengende Autoritäten entledigt und sei zum ungehemmten Toben in den Wald gegangen, um zu spielen, „Abenteuer zu erleben“ oder „Unsinn zu treiben“. Es scheint bedeutsam zu sein, dass dies unbeaufsichtigt geschehen konnte – ganz anders als „wie’s jetzt abläuft“. Daher irritiert an Cornelias Ausführungen, dass sie im unmittelbaren Anschluss an die zitierte Passage eine zu den Freiheitsentwürfen gegenläufige Erzählung vorbringt, denn „im Dorf hat natürlich jeder geguckt (.) alle haben auf alle aufgepasst (.) und die Omas im Neubau wussten dann schon, dass wir wieder scheiße gebaut haben“ und spätestens dann „haben’s die Eltern erfahren“. Die Kehrseite des Umstands, dass man gegenseitig auf einander Acht gegeben hat, bestand darin, dass man sich den nachbarschaftlichen Blicken nirgendwo vollends entziehen konnte. Der augenscheinliche Widerspruch zwischen den Unabhängigkeitsschilderungen auf der einen und der sozialen Kontrolle durch die Dorfgemeinschaft auf der anderen Seite tut hingegen Cornelias Kindheitsidealisierung und Freiheitsimagination keinen Abbruch. Ihre eigene Kindheit schildert Cornelia wie folgt:

wir haben (.) die Welt halt noch (.) eigenmächtig erkundet und (.) die Eltern waren noch nich überall hinterher; da hat auch nich ständig einer Angst gehabt und das betitele ich als vogelfrei

In Abgrenzung zu ihren Nichten, die, so lässt uns Cornelia wissen, infolge des überfürsorglichen Erziehungsstils ihres Bruders, den Bezug zur Natur verloren hätten, versucht Cornelia sich daran, auf den Begriff zu bringen, was sie darunter versteht, „vogelfrei“ zu sein. Entgegen dem eigentlichen Wortsinn erklärt Cornelia die Abwesenheit von Angst zu einem Kernelement der ‚Vogelfreiheit‘: Im Gegensatz zu heute sei ihre Elterngeneration weniger geneigt gewesen, „überall hinterher zu sein“ und sich zu sorgen – „die hatten keine Angst dass wir (.) uns verlaufen könnten oder dass wir weggefangen werden sowas gabs halt nich“. Cornelia leitet daraus ab, dass auch die Kinder „nicht so ne Angst“ haben mussten. Im eigentümlichen Gebrauch des Vogelfrei-Seins scheinen sich widersprüchliche Sinnebenen zu verdichten: Zum einen will sie damit einen Freiheitsdrang und -wunsch zum Ausdruck bringen, den sie in ihre Kindheit zurück projiziert, – frei sein, wie ein Vogel –, unwillkürlich deutet sie gleichsam an, dass die kindlichen Freiräume nicht nur positiv besetzt sind, beschreibt doch die Vogelfreiheit ihrem Wortsinn gemäß das Ausbleiben von Schutz und Fürsorge: Freisein im negativen Sinn.

Dieser doppelgesichtige Freiheitsbegriff wird auch an weiteren Szenen deutlich, in denen Cornelia zwischen den beiden in ihm angelegten Bedeutungsebenen regelrecht oszilliert. Auf das Beschwören von Unbekümmertheit und Eigenmächtigkeit folgt umstandslos, dass „natürlich strenge Regeln“ und ein „hartes Regime“ geherrscht hätten und den Kindern Respekt vor Erwachsenen und Hierarchien eingeflößt worden sei: „es war halt ganz ganz anders (.) es ist Hierarchie eingehalten worden, und man hat auf andere Weise rebelliert, und äh (.) aber so im Rahmen des Normalen des Kindsein“. Der „Rahmen des Normalen“, in dem die Kinder zu Zeiten der DDR rebellieren konnten, wurde durch das „harte Regime“ gesetzt und von der Dorfgemeinschaft exekutiert, indem sie anstelle der Eltern die Aufsicht übernommen hatten. Erscheint die kindliche „Horde“ dadurch gezähmt, rüttelt das dennoch nicht an Cornelias Selbstdeutung, denn dieser zufolge sei man „trotz dessen […] unbeaufsichtigt [gewesen] in der Zeit, wo man mit seinen Freunden unterwegs war“. Ihr Freiheitsbegriff kann vor diesem Hintergrund folgendermaßen übersetzt werden: „Autark“ zu sein, meint in diesem Zusammenhang, die Spielregeln – und auch die Schlupflöcher – zu kennen, deren Kenntnis es erlaubt, sich ‚frei‘ zu bewegen. Zu wissen, in der Enge der dörflichen Gemeinschaft überwacht zu werden, erlaubt es auch, sich darin zurechtzufinden: Die formellen und informellen Codes dieser Ordnung zeigen die bestehenden Handlungsspielräume an.

Mit den dörflich-autoritären Spielregeln verhält es sich demzufolge ähnlich, wie im Umgang mit staatlicher Repression in der DDR: „[M]an wusste halt was man sagen darf und was nicht (.) und es gab immer (.) zwei Sprachen […] und wenn sag ich mal ein (.) Staatssekretär anwesend war (.) dann wusste man halt was man sagen kann und was nicht“. Cornelia kommt u. a. deshalb zu einer durchwegs positiven Beurteilung ihrer Zeit im sozialistischen Osten, weil sie von den Repressionen laut eigener Angabe nicht betroffen gewesen sei – wer sowohl die offizielle als auch die inoffizielle Sprache fließend beherrschte, konnte sich mühelos durch die Strukturen manövrieren und sich schadlos halten.

### Konstruktionen natürlicher und unnatürlicher Ängste

Aus ihren Kindheitserfahrungen leitet Cornelia eine Angsttheorie ab, in der einerseits „natürliche“ „unnatürlichen“ Ängsten entgegenstehen, die sie jedoch andererseits im Interviewverlauf auch immer wieder mit Ideen rationaler bzw. irrationaler Ängste parallelisiert. Dieser Logik zufolge, das soll im Folgenden noch herausgearbeitet werden, sind natürliche Ängste gleichsam rational, „normal“ und „angemessen“, wogegen sich insbesondere in der Pandemie irrationale, überschießende Ängste manifestieren würden, die auf eine Entfremdung von natürlichen Gesetzmäßigkeiten verweisen.

Ihrer Selbstdarstellung zufolge neige Cornelia „überhaupt nicht zu Ängsten“, zumindest nicht zu unnatürlichen, und führt diese Eigenschaft auf die ihr als Kind zugestandenen Freiheitsgrade zurück:

also wenn Not da is (.) hab ich natürlich auch ne lebenserhaltende Angst (.) aber nich so übertrieben, wies jetzt is (.) diese Angst vor Erkrankung vor Keimen vor Bakterien das is völlig unnatürlich und dazu neig ich nich und ich glaube das is in meiner Kindheit begründet […] dieses ganze übertriebene in Angst (.) gebadete (.) gabs bei uns nich

Entgegen ihrer Behauptung nur „lebenserhaltende Ängste“ zu verspüren, deuten sich in ihren Kindheitserzählungen an mehreren Stellen durchaus auch latente, diffuse – stellenweise auch als ‚irrational‘ gewertete – Ängste an, etwa als darin ein „beigefarbener Lada“ erscheint, mit dem Phantasien verknüpft werden, „weggefangen“ werden zu können. Irrationale Ängste schreibt Cornelia jedoch manifest nur den sog. „Sagrotaneltern“ zu: An diesen beklagt sie, dass sie „mehr darauf achten, dass ihre Kinder desinfiziert sind“, als anzuerkennen, dass „so ein Immunsystem trainiert werden will“. Indem Kinder im Wald „toben“ und mit Schmutz in Kontakt kommen, ohne unter der immunisierenden Aufsicht der Eltern zu stehen, werden sie zur Widerstandsfähigkeit erzogen. Das Spielen im Wald und Unfugtreiben ist demzufolge nicht mehr Selbstzweck, sondern dient der Vorbereitung auf künftige Widrigkeiten. Nicht nur der mangelnde Kontakt mit Schmutz durchkreuzt die gesunde Entwicklung des Immunsystems, die den Kindern „eingeimpften Ängste“ schwächen es darüber hinaus. Demgegenüber habe sie selbst einen „natürlichen Bezug zur Natur“, und weil sie „nicht so ängstlich und empfindlich“ sei, habe sie auch „so ein gutes Immunsystem“.

weil das (.) damals einfach noch anders war (.) und weil äh wir un- (.) weil wir halt (1) äh wie soll ich sagen das Ergebnis unsrer Handlungen (.) am eigenen Leib spüren konnten; (.) weil wir so frei waren (1) denkt man natürlich nach; und (.) da trampelt man nich aufn morschen Ast, weil man logisch denkt (.) und äh ja (.) ich denke das ist dem geschuldet.

Man könnte Cornelias Selbstbeschreibung wie folgt übersetzen: Einen natürlichen Bezug zur Natur zu haben, bedeutet die Natur und ihre Gesetzmäßigkeiten zu kennenund sich frei in ihr bewegen zu können. Entlang dieser natürlichen Gesetzmäßigkeiten und in Abgrenzung von unnatürlichen, lähmenden Ängsten und unzulässigen Eingriffen von außen, entwirft sie implizit Vorstellungen dessen, was wir als ‚natürliche Ordnung‘ bezeichnen würden. Diese Ordnung zu begreifen, erfordert ‚natürlich‘ nachzudenken und die logischen Schlüsse aus dem eigenen Handeln zu ziehen: Wer einmal auf einen morschen Ast tritt, tut das kein weiteres mal. Um dieses natürliche Regelwerk logisch durchdringen zu können, musste man es hingegen zunächst „am eigenen Leib spüren“. In dieser kurzen Passage laufen mehrere Bedeutungsebenen zusammen: Wie die dörflichen Handlungsdispositive gilt es auch, die Naturgesetze ‚logisch‘ zu durchdringen und sich in ihnen zurechtzufinden. Erreicht werden kann das allerdings nur handlungspraktisch, indem man die ‚unnatürliche‘ Angst ablegt und sich in diese Strukturen hineinbegibt. Diese ‚irrationale‘ Spielart der Angst abzustreifen, oder sie gar nicht erst an sich heranzulassen, bedeutet aber auch, sich potenziell gefährlichen Situationen auszusetzen und sich durch die Erfahrungen und möglicherweise am eigenen Leib erfahrene Schmerzen – sie spricht an einer anderen Stelle davon, dass sich die Eltern natürlicherweise Sorgen darum machen, dass die Kinder im Wald den Arm brechen – für künftige Hindernisse zu wappnen.

Hinweise auf Verletzungen und Kränkungen finden sich in Cornelias Ausführungen auch an anderer Stelle, etwa wenn sie näher auf die Beziehung zu ihrem Vater eingeht: Dieser sei in der Stadt wegen ihres „besonderen Styles“ mitunter einen Meter hinter ihr gegangen, weil er sich für seine Tochter geschämt habe. Doch auch diesen Demütigungen kann Cornelia nachträglich etwas Gewinnbringendes abringen, denn im Gegensatz zu ihr selbst habe ihr Vater erkannt, dass sie sich mit ihrem Auftreten selbst im Weg gestanden sei. Die versöhnliche Einstellung scheint ihr als junges Mädchen hingegen noch nicht vergönnt gewesen zu sein: Cornelia deutet an, dass sie damals durchaus „wütend oder gegrämt“ gewesen sei, heute kehrt sie diese Situationen in die „beste Vorbereitung fürs Leben“ um. Nüchtern betrachtet, losgelöst von allen irrationalen Gefühlen, so könnte man übersetzen, ist ihr Vater im Recht gewesen, als er sie auf die sozialen Spielregeln hingewiesen hat. Die Szene ist aber noch aus einem weiteren Grund instruktiv, denn Cornelia leitet diese ein, indem sie sich als „rebellierend“ und als junge Frau darstellt, die „Grenzen ausgetestet“ hat. Diesen Widerstandsgestus verwirft Cornelia nicht, sondern lädt auch ihn mit Zweckrationalität auf: Die Rebellion gegen das mit den Eltern assoziierte „spießige Dasein“ und ihr Widerwillen sich diesem zu fügen, wird selbst wiederum zu einer Lektion erklärt, die sich durch die Widrigkeiten des Lebens rechtfertigt. Die ‚Rebellion im Rahmen des Normalen‘ besteht demnach darin, Grenzen auszutesten und normative Maßstäbe in Frage zu stellen, doch nicht primär, um sie zu modifizieren, sondern um an den Widerständen zu wachsen und für künftige Hindernisse gerüstet zu sein.

Demgegenüber könne Cornelia an manchen Freund:innen beobachten, welche Folgen es haben könne, wenn man „sehr sehr weich und liebevoll erzogen“ wird:

die kommen mit Stresssituationen überhaupt nich klar (1) das is (.) das is verrückt, also das (.) also mir tuts manchmal n bisschen leid, die erscheinen mir regelrecht (.) unbeholfen (.) weil sie mit Widerständen oder mit Druck und Stress (.) nicht gut aus- (oder) umkommen.

Die Kehrseite einer liebevollen Erziehung besteht im mangelhaften Rüstzeug, um mit Druck- und Stresssituationen zurecht zu kommen, die so überwältigend werden können, dass man nicht nur überfordert sei, sondern gar „umkommen“ könne. Die Außenwelt wird als derart bedrohlich wahrgenommen, dass man Todesgefahr nur abwenden kann, indem man sich bereits früh abhärtet.

Auf Grundlage der bisher analysierten Passagen inszeniert sich Cornelia auf einer *manifesten Ebene* als freiheitsliebende und widerständige Person, die in Resonanz mit einer ‚natürlichen‘ Ordnung lebt, die sie durch alltagspraktische Aneignung durchschaut zu haben meint. Darunter fallen auch das implizite dörfliche Regelsystem sowie das DDR-Staatssystem, in dem sie gelernt hat, sich trotz allgegenwärtiger sozialer Kontrolle ‚frei‘ zu bewegen. In diesen Ordnungssystemen verortet sie eine stabilisierende Gesetzmäßigkeit, auch wenn sie bisweilen wiederum als einschränkend erlebt wird. Die Erziehungspraktiken ihres Vaters – von ihrer Mutter erfahren wir kaum etwas – adelt sie zur vorausschauenden Vorbereitung auf die Widrigkeiten des Lebens, wobei sie durchaus auch zu erkennen gibt, dass sie erst mit etwas Abstand zu dieser Betrachtungsweise kommen konnte. Die abhärtenden Erfahrungen in der Natur und durch die Disziplinierungen des Vaters münden ihrer Selbstdeutung zufolge darin, dass sie sich von irrationalen und lähmenden Ängsten befreien kann und dadurch wird ihr ‚rationaler‘ Blick auf die Welt erst möglich. *Latent *gemacht werden muss hingegen das Einengende der von Cornelia beschworenen Dorfgemeinschaft und Gesetzmäßigkeiten sowie das Bedrohliche der ungezähmten Natur. Sowohl in der Passage mit ihrem Vater, der ihren „Style“ so gar nicht akzeptiert, wie auch in ihren Erzählungen über „das strenge Regime“, in der sie auf den in den Nachkriegszeit neuaufgelegte NS-Ratgeber *Die deutsche Mutter und ihr erstes Kind* als Leitlinie der elterlichen Erziehung hinweist, ein „Folterbuch“, wie sie sagt, verweist sie auch auf Ängste und Verletzlichkeiten und letztendlich auch auf Bedürfnisse nach Schutz und Fürsorge – auch wenn sie im Nachhinein das Regime doch wieder als „immer konstruktiv“ und das Verhalten des Vaters als eines „zu meinem Schutz“ beschönigt. Die Spannung zwischen manifesten und latenten Sinnebenen wird u. a. durch die Phantasien deutlich, beim Toben im Wald der Gefahr ausgesetzt gewesen sein zu können, vom jemandem weggefangen werden zu können.

### Zäsuren innerer und äußerer Ordnung

Die Einsichten in die Gesetzmäßigkeiten der Gesellschaft erweisen sich jedoch als prekär und von zersetzenden Kräften bedroht. Als wegweisend beschreibt Cornelia dabei allen voran zwei gesellschaftliche Umbrüche, die sie in ihren Erörterungen wohl nicht zufällig parallelisiert: die deutsche Vereinigung und die Corona-Krise. Nach dem Zerfall der DDR sei für ihren Vater „eine Welt zusammengebrochen“, unter deren Trümmern er wie so viele andere „zerbrochen“ sei. Auch für Cornelia sei die Wende entscheidend gewesen, weil mit ihr materielle Prinzipien Einzug gehalten hätten und plötzlich nicht mehr wichtig gewesen sei, welche „Fähigkeiten und Fertigkeiten“ man besetzt, sondern welche Marken man trägt. Dass „die DDR an den Westen verkauft“ worden sei, und zwar „auf ganz berechnende asoziale Weise“, ohne an Einzelschicksale zu denken, ruft bei Cornelia Erinnerungen an die Pandemie wach:

und es is ja jetzt ähnlich, es werden Entscheidungen getroffen, von Menschen, die finde ich die die (.) Haftung zur Realität oder zum normalen Boden zum Bürger verloren haben (.) die wissen gar nich wies bei manchen Leuten aussieht.

Während Cornelia im Interview Ängste vor dem Virus als „unbegründet“ hinstellt, sieht sie die aktuelle Problemsituation in den „idiotischen Entscheidungen“ der politischen Führung, die „viel Menschenleben“ gekostet und auch verhindert hätten, dass schnell eine „Herdenimmunität“ entstehe. Auch hier scheint Cornelia eine sich selbst regulierende Natur vorauszusetzen, während es die Regulierungsversuche der Politik sind, die die Naturgesetze ins Wanken bringen.

Auch der „freie Markt“ habe seine Gesetzmäßigkeiten, die offenbar nicht beeinflusst werden sollten. „Berufspolitiker“ seien schlichtweg nicht dazu in der Lage adäquate Strategien zu entwickeln, weil sie sich – im Gegensatz zu Cornelia selbst – nie auf dem „freien Markt“ durchsetzen mussten. Auf dem Markt herrschen jene Naturgesetze, gegen die man sich behaupten und an denen man sich stählen muss. Wer diese Erfahrungen nicht gemacht hat, dem ist es erst gar zuzutrauen, Maßnahmen zu ergreifen, die in Einklang mit eben diesen Gesetzmäßigkeiten stehen. Die natürlichen Selektionsprozesse des freien Marktes hingegen sind durch künstliche Einflussnahme bzw. die Verschränkung von Wirtschaft und Politik außer Kraft gesetzt, wie uns auf dem Weltwirtschaftsforum (WEF) vorgeführt worden sei:

das es so ne Verein gibt (.) völlich rational, die wollen sich vernetzen, die wollen sich gegenseitig stärken, die wollen den Markt aufteilen versteh ich, aber frag ich mich (.) was haben denn da Leute wie Angela Merkel […] was haben denn (.) so ne Leute bei so ner Lobbyveranstaltung zu suchen (.) versteh ich nich (.) denn eigentlich ist doch unsre Regierung die Vertretung der Bürger (.) also des Volkes (2) so erscheint es mir aber als wäre unsre Regierung die Vertretung der Industrie.

Cornelia beklagt nicht, dass sich Unternehmer:innen vernetzen, gegenseitig den Rücken stärken und sich den Markt aufteilen, sondern die Anwesenheit hochrangiger Politiker:innen. Die Regierung solle nicht als Vertretung der Industrie fungieren, sondern die freien und ‚natürlichen‘ Marktgesetze wirken lassen, und ihre Bürger auf die Verwerfungen eben dieser Kräfte vorbereiten, anstatt Angst zu schüren und ‚unnatürlichen‘ Entwicklungen Vorschub zu leisten. Die politischen Einflussnahmen, so können wir Cornelias Ausführungen deuten, untergraben den „natürlichen Bezug zur Natur“ – emblematisch dafür steht das WEF, auf dem „Eliten“ hinter dem Rücken der Bevölkerung Pläne schmieden würden, die „völlig wider die Natur“ seien.

Die Entfremdung von der Natur zieht verheerende Konsequenzen nach sich: Es entstehen verängstigte Individuen, die mangels Abhärtung zerbrechen, und nicht in der Lage sind, die Irrationalität des Pandemiemanagements zu durchschauen. Ganz wie ihre liebevoll erzogenen Freundinnen, sind die als wehrlos imaginierten Opfer der Schutzmaßnahmen auf Cornelias Protest angewiesen: „[M]anchmal ist es auch (.) finde ich ne Form von Bürgerpflicht (.) sich auch manchmal einzumischen; auch wenn’s einen nicht betrifft, wenn man Unrecht sieht“. Im „Widerstand“ befindet sich Cornelia nicht ihrer selbst wegen, sei sie doch von der Krise „bis auf das Berufsverbot nicht weiter [betroffen]“, sondern um ihrer moralischen Verpflichtung gegenüber den sozial Schwachen nachzukommen. Über das wirkliche Ausmaß von Cornelias Betroffenheit können wir hier nur spekulieren, ihrer Selbstinszenierung zufolge jedoch haben die sozialen Konsequenzen anscheinend einen Bogen um ihr Refugium gemacht, das sie sich in ihrem Zuhause eingerichtet hat, auf dem sie ihr Leben „naturverbunden und umweltbewusst“ verbringt. Einerseits beschwört Cornelia demnach Bilder gesellschaftlicher Zäsuren und von der Zerrüttung bestehender Ordnungssysteme durch die Wende wie auch durch die Pandemie, andererseits sind die Betroffenen stets die anderen: ihre Eltern, Freundinnen, die Kinder oder die ‚Schwachen‘. Ihr Widerstand beschränkt sich auf verbale Bekundungen, auf unregelmäßiger Protestteilnahme in der Nähe ihres Heimatorts – Massenprotesten gegenüber ist sie skeptisch eingestellt – und in der Parteinahme für andere, bspw. wenn sie eine verängstigte Freundin zu beruhigen versucht, indem sie in nächtlichen Rechercheaktionen Informationen über die ‚wirkliche‘ Beschaffenheit des Virus zusammenträgt, um mit ‚irrationalen‘ Ängsten aufzuräumen: „[Ich] hab dann meine Freundin darüber informiert (.) hab gesagt hör dir das doch mal an und lies dir das doch mal durch und das hat sie gemacht und damit war die Angst vorbei“. Dass Cornelia durch diese rationale Herangehensweise nicht nur anderen, sondern auch sich selbst die Angst nimmt, deutet sie nur wenige Zeilen weiter an:

ich bin nicht gut in Angst zu versetzen; das hat noch nie geklappt; weil ich da immer bissl analytisch rangeh und erstmal sage moment mal (.) so und so is es (.) wie kann das kommen, woher, was steht damit in Verbindung, wie setzt sich das durch (.) und wenn ich dadurch Sachen so analysiere; is ja gar keine Zeit für Angst und Panik.

Indem Cornelia sich akribisch auf die Suche nach den Ursachen und Eigenschaften des Virus macht – dabei macht sie klar, dass sie der Presse grundsätzlich nicht mehr vertraue und viele medizinische Studien und ebenso die meisten Corona-Maßnahmen „unsinnig“ seien – und Verbindungen freilegt (mehrmals betont sie, dass sie keine Verschwörungstheoretikerin sei, diese aber häufig nicht unrecht hätten), bleibe auch ihr keine Zeit (mehr) für Angst und Panik. Es erscheint daher nicht so, als hätte es „noch nie geklappt“ Cornelia Angst einzuflößen, jedoch helfen die Recherchen und ihr ‚rationales‘ Vorgehen im Allgemeinen eine stringente Logik in der Wirklichkeit zu erkennen und dergestalt eine destabilisierte innere wie äußere Ordnung (wieder-)herzustellen.

Dass die in Cornelias Innenleben aufgebaute Ordnung durch die Krise brüchig wird, lässt sich auch an weiteren Transkriptstellen veranschaulichen. Wir können bspw. sehen, dass sich ihre an der Maskenpflicht und der Impfung entfachende Sorge um das Wohl der Kinder auf eine Art und Weise Bahn bricht, die ihren kindlichen Freiheitsentwürfen diametral entgegensteht:

also ich bin froh dass ich keine Kinder hätte ich wär wahrscheinlich jetzt im Knast (.) weil wenn ich Kinder habe (.) dann bin ich glaub ich auch ne Helikoptermutter dann (.) ich bin ja (.) ich hab die Verantwortung über die (.) es is doch meine Aufgabe als Elternteil zum besten Wissen und Gewissen meine Kinder zu schützen; egal vor was und auch vor Maßnahmen die nich gesund sein können.

Entgegen ihrer affirmierenden Haltung zum bisweilen vernachlässigenden Erziehungsstil ihrer Eltern, der im Gegenzug mit vielen Freiheitsgraden verbunden gewesen sei, macht sich Cornelia vor dem Erfahrungshintergrund der Pandemie für eine durch allumfassenden Schutz gekennzeichnete Erziehung stark. Das Virus stellt damit weniger eine Gefahr, sondern vielmehr eine Bewährungsprobe unter vielen dar, an denen Kinder und ihre Immunsysteme wachsen können. Die eigentliche Bedrohung geht auch hier wieder von den als ‚unnatürlicher‘ Eingriff beschworenen Maßnahmen der Regierung aus, allen voran der Maskenpflicht, die sie dazu veranlasst, mit ihren manifesten Überzeugungen zu brechen: Plötzlich entwirft Cornelia ein Szenario, in dem sie selbst zur „Helikoptermutter“ wird, die ihre fiktiven Kinder nicht aus den Augen lassen würde, um sie vor schädigenden Eingriffen zu schützen. An den Herausforderungen, die die Corona-Maßnahmen mit sich bringen, können die Kinder offenbar nicht wachsen, sondern an ihnen gehen sie zugrunde – die Masken würden die vom „Kindergehirn“ benötigte Menge an Sauerstoff abschnüren. Überdies hätten die Maskenaffären um Jens Spahn und Markus Söder bewiesen, dass diese nicht dem Gesundheitsschutz, sondern den Profitinteressen von mit der Wirtschaft verbandelter Politiker dienen – die berechtigte Kritik an der Bereicherung durch Politiker:innen in der Krise wird hier eindeutig verschwörungsideologisch überformt. Kurzum: Im Anbetracht des Angriffs auf die ‚natürliche Ordnung‘ durch korrumpierte und klandestine Mächte, deutet Cornelia die Abkehr von den Freiheitsidealen ihrer Kindheit zur *rationalen* Reaktion auf ‚künstliche‘ Einflussnahmen um.

Manifest inszeniert sich Cornelia demnach als furchtlose Widerstandskämpferin, die sogar bereit wäre in den „Knast“ zu gehen, um ihre moralische Pflicht zu erfüllen: den Schutz der sozial Schwachen auf der einen und die Bewahrung dessen, was wir als auf natürlichen Gesetzmäßigkeiten beruhende Ordnung beschrieben haben, auf der anderen Seite. Latent zeigen sich hingegen auch Ängste vor dem Verlust eben dieser haltgebenden Struktur, die immer wieder bedroht wird – sei es durch die Wende, in der das Wertesystem der DDR durch Konsumismus und Individualismus abgelöst wurde, oder das Pandemiemanagement. Letzteres offenbart, wenn man Cornelias Ausführungen folgt, die Konfundierung von Politik und Wirtschaftsinteressen, die auf dem WEF auf die Spitze getrieben werde. Grenzen lösen sich auch zwischen Nationen und Ethnien auf: Es sorgt bei Cornelia für Verunsicherung, dass Menschen in den Westen „gelotst“ würden, obwohl sie da „teilweise gar nicht reinpassen und auch nicht reinpassen möchten“. Der dadurch entstehenden inneren – und der imaginierten sozialen – Unruhe versucht Cornelia durch ‚rationale‘ Analysen Herr zu werden. Erst diese Analysefähigkeit erlaubt es ihr die ‚irrationale‘ Angst abzustreifen bzw. sich diese vom Leib zu halten und die Mächte zu erkennen, die sich gegen die Natur verschwören und nicht nur äußeres, sondern auch inneres Chaos anzurichten drohen.

## Diskussion

### Fragmente der Conspirituality

Manifest beschreibt die Interviewte eine aktuelle chaotische Situation, die im Zusammenhang einer Verfallsgeschichte steht. Die in der Kindheit im Heimatdorf erlebte harmonische Ordnung wird durch Wende und Corona-Krise bedroht. Cornelia sieht in der Gesellschaft eine Vermischung der Praktiken verschiedener Sphären – sie benennt Volk, Politik und Wirtschaft –, die getrennt bleiben sollten und deren Vermischung als gefährlich einzuschätzen sei. Im Zentrum steht dabei die Verbreitung von materiellen Werten, wie Profitorientierung und Warenfetisch, in Politik und Volk, die mit dem Ende der DDR einsetzt und die Vermischung der Sphären vorbereitet hätten, da wirtschaftliche Prinzipien nun vermehrt Einzug in politische Entscheidungsprozesse zu nehmen scheinen. Diese Trennung der drei Sphären irritiert zuerst, wird jedoch verständlich, wenn man in den Blick nimmt, dass ihre Kritik dabei die Dreigliederung des sozialen Organismus von Rudolf Steiner implizit adaptiert. Rudolf Steiner, Begründer der Anthroposophie, ist eine bedeutsame Figur in der Geschichte der westlichen Esoterik, im Besonderen im deutschsprachigen Bereich (Leijenhorst 2006, S. 89; McDermott 2016, S. 264). Im Folgenden wollen wir anhand der Kernthesen des Steiner’schen Werks als hermeneutischer Brille ergründen, wie sich Cornelias Weltbild entlang dessen Modells der Dreigliederung aufspannt. Obgleich die Bezüge in der Erzählung nicht explizit gemacht werden, und wir nicht wissen, ob sie Steiner studiert hat, sind die Parallelen augenfällig. Es lohnt sich daher ein Blick auf Cornelias Aneignung anthroposophischer Ideen vor dem Hintergrund ihrer Biografie und ihrem Krisenerleben, und darauf, welche Funktion damit erfüllt wird. Dadurch lässt sich in einem nächsten Schritt verstehen, welche Funktion mit den Versatzstücken der conspirituality erfüllt wird. Die Dreigliederung in Geistesleben, Rechtsleben und Wirtschaftsleben bildet das Kernstück der Steiner’schen Soziallehre, die nicht in geschlossener Form, sondern lediglich fragmentarisch vorliegt (Luttermann 1990, S. 9). Dies liegt mitunter daran, dass diese für eine politische Praxis formuliert wurde (Steiner 1982a, S. 11 ff.; Zander 2019, S. 174 f.).^3^ Eine systematische Darstellung kann somit auch nur bedingt die Rezeption der Soziallehre Steiners beleuchten, deshalb dient sie hier lediglich als Modell zum Verständnis und der Einordnung der Positionen der Interviewten.

Steiners Soziallehre stellt eine spirituelle Form des Antikapitalismus dar, die er in Abgrenzung zum Marxismus entwickelt. Diesem stellt Steiner seine Einsicht in die soziale Dreigliedrigkeit entgegen, die jeden der Bereiche sich frei nach seinen eigenen Dynamiken entfalten lässt. Die esoterische Grundlage der sozialen Dreigliederung ist dabei, dass eine „Oberhoheit des Geistigen“ (Zander 2019, S. 175) bestehe. ‚Richtige‘ soziale Praxis könne dabei nur von jenen beschrieben werden, die Einblick in den neuen Geist haben und sich von ihrer ‚Verblendung‘ befreien können, die das Proletariat und dessen Führer prägt, die nur Wirtschaftsleben oder Rechtsleben bzw. Politik berücksichtigen (Steiner 1976, S. 41, 47; Steiner 1982a, S. 53 ff., 81 f.; Zander 2019, S. 175). Außerdem finden sich in Steiners Ausführungen zur Dreigliederung wiederholt Bezüge auf die esoterische Geschichtsschau (Steiner 1982a, S. 25, 61 f.). Daraus ließen sich aktuelle geschichtliche Anforderungen an das Bewusstsein herauslesen und in eine fortschreitende Menschheitsentwicklung einordnen. Die Fortschrittsbewegung sei bereits im Altertum mythisch angelegt gewesen und setze in den Epochen gewisse Entwicklungskräfte frei, welche dann bestimmte Entwicklungsschritte hervorbrächten (Luttermann 1990, S. 125 ff.; Martins 2012, S. 123). An deren Ende stehe eine *menschliche *Welt, die den sozialen Organismus analog zum Menschen, entsprechend der Dreigliederung des Leibes in Kopfsystem, Kreislaufsystem Stoffwechselsystem, durchbilde (Steiner 1976, S. 26, 58, 61 ff.).

Auffällig ist, dass sich die Interviewte lediglich auf Versatzstücke der Steiner’schen Theorie bezieht, die in die parallelisierte Beschreibung des eigenen Aufwachsens und der Situation während der Corona-Pandemie eingeflochten werden. Verstanden werden könnte dieser in Teilen affirmierende Umgang mit theoretischen Versatzstücken im Kontext der eingangs beschriebenen Dynamik der „Occulture“ (Asprem und Dyrendal 2015, S. 368; Partridge 2014), in der unterschiedliche Momente esoterisch-spirituellen Denkens eine Popularisierungsbewegung durchlaufen und anschließend von den Individuen ohne die Notwendigkeit eines geschlossenen esoterischen Weltbildes angeeignet werden können. Letztlich bleibt jedoch unklar, ob diese esoterischen Versatzstücke aus einer aktiven Auseinandersetzung mit dem Werk Steiners hervorgeht oder diese als Fragmente im Sinne der Occulture unabhängig vom Werk aufgenommen wurden. Die soziale Dreigliederung scheint für sie ein Modell der ‚natürlichen Ordnung‘ der Gesellschaft darzustellen und erscheint als Ziel und Utopie ihres Plädoyers für ein harmonisches Verhältnis von Mensch und Natur (Palmisano und Pannofino 2021).

Die Fortschrittsmetaphysik Steiners zeigt die Interviewte allerdings nicht. Vielmehr tritt ihr Herkunftsdorf in ihren Kindheitserzählungen als Bild dieser harmonischen Dreigliederung in Erscheinung. Rechtsleben und Politik sind außen vor gelassen und treten nur im Bild des ‚Ladas‘ eindeutig in Erscheinung, der mit einer diffusen angstvollen Phantasie verknüpft wird, „weggefangen“ zu werden. Von diesem abgesehen können sich die Kinder frei von politischen oder wirtschaftlichen Imperativen entwickeln, wodurch ihr Geistesleben sich unabhängig entfalten und die darin gründenden Kräfte in das Wirtschaftsleben eingebracht werden können (Steiner 1982a, S. 33). In ihrem Heimatdorf zeigt sich das Wirtschaftsleben im lokalen Handwerk, in welchem sich die Kinder ausprobieren können und dort „Fähigkeiten und Fertigkeiten“ erwerben. Ihre eigene Bildungs- und Berufsbiographie ist dann auch geprägt davon, sich nach ihren Talenten einzubringen und keinen ökonomischen oder politischen Zwängen zu unterliegen. Diese Darstellung begründet sowohl ihr „natürliches Verhältnis zur Natur“ als auch ihr persönliches Leben in Einklang mit der Natur selbst. Mit ihrem eigenen spirituellen Zugang zur Natur und ihrem Leben als Teil eines natürlichen Gefüges markiert sie im Anschluss ihre besondere Erkenntnisposition, von der aus sie soziale Prozesse bewerten kann. Dies erinnert an die Darstellung Steiners der inneren Wesenheit des Naturwirkens im menschlichen Organismus und dessen zumindest instinktive Erkenntnis der Notwendigkeiten des sozialen Organismus, der das Denken nachstreben soll (Steiner 1976, S. 59). Die Organisation des sozialen Organismus ergibt sich aus dem ‚gesunden‘ Empfinden des Menschen, das sich aus der Betrachtung der Natur ergebe, so schreibt Steiner (ebd., S. 60), wodurch die Trennung von Verstand und Natur des modernen, westlichen Individuums (McDermott 2016, S. 268) überwunden werden soll. Auch für die Interviewte ist die Natur Lehrmeisterin für das naturgemäße Verhalten im Sozialen – in ihr soll man Toben können, um sich das nötige Rüstzeug für die zu erwartenden Widrigkeiten anzueignen. Steiner formuliert daraus auch die Aufgabe der schulischen Bildung (Steiner 1976, S. 61).^4^ Das rechte Empfinden ist bei der Interviewten das natürliche Verhältnis und die rationale Angst bzw. der Respekt vor der Natur, wodurch auch Stress und Krankheit vermieden werden. Die Natur bildet bei Steiner die Grundlage aller Sphären des sozialen Organismus (ebd., S. 64 ff.). Das sieht die Interviewte in der DDR-Zeit verwirklicht. Mit dem Ende der DDR entfremdet sich die Gesellschaft von ihren Naturgrundlagen und statt um Fähigkeiten und Talent geht es nur noch um Besitz. Die Menschen würden sich ausschließlich an materiellen Interessen orientieren (ebd., S. 25), das Wirtschaftsleben kolonialisiert das Geistesleben und mit der Corona-Pandemie kolonialisiert zusätzlich das Rechtsleben das Wirtschaftsleben, wie Cornelia an der Umwandlung der Politik zur ‚Lobbyveranstaltung‘ kritisiert. Hier tritt zu ihrer spirituellen Vorstellung von der natürlichen Ordnung sozialer Systeme und ihren Relationen eine implizite Verschwörungsideologie, die den erklärenden Hintergrund beschafft und die Verfallsgeschichte einleitet, die sich als Prozess der Korrumpierung ihrer Mitmenschen lesen lässt – der Verfall wird bei ihr so zu einem Intentional. Ihr Verschwörungsdenken dreht sich dabei um die bewusst herbeigeführte Entfremdung von ‚der Natur‘, der eine, unter Bezugnahme auf Versatzstücke der Steiner’schen Theorie entwickelte ‚richtige‘ und während ihrer Kindheit bereits verwirklichte Einrichtung der Welt gegenübergestellt wird. So werden von ihr zum einen die Maßnahmen und ein Kult des Konsums als Treiber der Entfremdung ausgemacht, jedoch versucht sie darüber hinaus konkrete Gruppen, wie das WEF oder korrumpierte Politiker:innen zu benennen. Sie entfaltet hier eine Erzählung von (mächtigen) Gruppen an der Schnittstelle von Politik und Wirtschaft, die mit ihrem Handeln das Gleichgewicht des Sozialen bezogen auf die verschiedenen Sphären durcheinander bringen. Implizite Anleihen nimmt sie dazu bei der Verschwörungsideologie die um den Vorschlag des ‚Great Reset‘ herum entstanden ist. Dieses Narrativ postuliert, dass die Corona-Pandemie von globalen Eliten erzeugt wurde, um die Herrschaft über die globale Ökonomie zu erlangen (Koblentz-Stenzler und Pack 2021). In der Vorstellung Cornelias führt eben diese Verschwörung zur Übernahme der globalen Welt zu einer Intensivierung des Verfallsprozesses und zu einer fortschreitenden Erosion der natürlichen Dreigliederung sozialer Systeme. Hier wird augenfällig, wie sich im Kontext von conspirituality Verschwörungsideologien mit Spiritualitätserzählungen amalgamieren und als dieses Amalgam die Funktion eines reaktionären Welterklärungsversuchs erfüllen.

Die Verstöße gegen die natürliche Dreigliederung des sozialen Organismus und die damit einhergehende Gefährdung des Geisteslebens bzw. in Cornelias Benennung des Volkes und dessen freier Entfaltung sowie des Wirtschaftslebens, welches des freien Geisteslebens bedarf, werden den Menschen als Übertritte nicht einmal mehr bewusst. Hier tauchen bei Cornelia die „Sagrotaneltern“ als Agenten der Entfremdung auf, die jeglichen Kontakt der Kinder mit Dreck und Bakterien und damit mit Natur unterbinden. Im Angesicht der Gefahr und der insbesondere mit der Impfung in Zusammenhang gebrachte Bedrohung der Kinder im Zuge der Corona-Maßnahmen unterstützt sie dann selbst stark eingreifende und regulierende Eltern, die die Bedrohung für eine gesunde kindliche Entwicklung verhindern müssen. Die zentrale Bedrohung findet sich bei ihr in der Vermischung der Sphären, die die natürliche Ordnung des sozialen Organismus gefährdet, was wie bei Steiner nur zu einer Katastrophe führen kann (Steiner 1976, S. 24, 1982a, S. 16 f.), die sich in „Geburtenkontrolle“, Nanotechnologie u. a. bereits ankündigt. Durch die Entfremdung von der Natur entstehe eine unnatürliche Angst vor der Natur, welche durch rigide Hygienemaßnahmen ganz aus dem Leben verdrängt wird. Diese unnatürliche Angst, die Cornelia ebenfalls zum Thema macht, ermöglicht ein Ineinanderfallen von Wirtschafts- und Rechtsleben, da das Geistesleben nicht mehr als Gegengewicht funktioniert. Letztlich, so Cornelia, sei nicht die Natur zu fürchten, sondern die Schädigungen stammen allesamt aus den Maßnahmen zu ihrer Eindämmung („Erhöhung des Totraum“ durch die Masken), die darüber hinaus wirkungslos sind. Warnende Stimmen würden ignoriert, diskreditiert und als rechtsextrem diffamiert.

### Natur als „Schiefheilungsschablone“

Ausgehend von der Bestimmung der spezifischen Verzahnung von Verschwörungsdenken und esoterisch-spirituellen Ideologiefragmenten sowie den impliziten Bezügen auf die Lehren Steiners im vorgestellten Fall, wollen wir uns den latenten Dynamiken und dem Moment der conspirituality widmen.

Das Weltbild der Interviewten stützt sich auf einen imaginierten harmonischen Naturzustand, der in die frühe Kindheit zurückprojiziert wird und in der Phantasie besteht, sich frei von den Zugriffen der Eltern wie der politischen Sphäre entfalten zu können. Die ‚natürliche Ordnung‘ wird nicht nur von einer gezähmten äußeren Natur verkörpert, mit der man in Einklang leben solle, sondern gleichsam von einer ‚natürlichen‘ (Dorf‑)Gemeinschaft in der DDR, in der aufeinander Rücksicht genommen und Acht gegeben wurde. Die Interpretation des Interviews hat jedoch zutage gebracht, dass quer zu den Entwürfen einer unbeschwerten und mit reichlich Freiheitsgrade ausgestatteten Kindheit teils konkrete, teils diffuse Ängste und Bedrohungslagen sowie normierende und disziplinierende Zugriffe stehen: Davon zeugen Schilderungen des „strengen Regimes“ der Eltern, die Demütigungen durch den Vater oder die von der Dorfgemeinschaft ausgeübte soziale Kontrolle, die zwar einerseits Aufsichts-, andererseits jedoch Disziplinierungsfunktionen erfüllte. Gegenläufige Bilder werden aber auch durch die Gefahr, „weggefangen“ werden zu können, aufgerufen. Diese Gefühlslagen, die eigentlich die vorgebrachte Idylle stören würden, werden entweder dadurch rationalisiert, dass sie als notwendiger Teil der Naturordnung einen positiven Sinn erhalten: Sie stärken Körper und Psyche gegen zukünftige Krisen. Vor allem aber werden sie projektiv in Entfremdungserzählungen und Verschwörungsideologien abgewehrt. Diesen Vorgängen wollen wir im Folgenden genauer nachgehen.

In den esoterisch-spirituellen Wahrnehmungsmustern der Interviewten spaltet sich die Welt in eine ‚rationale‘ und wohlwollende Natur bzw. Gemeinschaft sowie in ‚unnatürliche‘ bzw. irrationale Abirrungen, die unentwegt drohen, erstere aus dem Gleichgewicht zu bringen (vgl. Schließler et al. 2020, S. 293; sowie die Ausführungen zur conspirituality und zu Steiner im vorigen Abschnitt). Es lohnt sich, diese Projektionsdynamiken vor dem Hintergrund dessen zu beleuchten, was in der psychoanalytischen Sozialpsychologie unter dem Begriff der *Schiefheilung *(Brunner 2016; empirisch Knasmüller und Brunner 2022) verhandelt wird: Das Konzept beschreibt, wie innere Konfliktlagen als Teil einer durch das Band eines ‚kollektiven Symptoms‘ verbundenen Gemeinschaft bearbeitet werden können, indem ambivalente Regungen und Wünsche sowie Aggressionen, Ängste und andere als störend empfundene Affekte projektiv ausgelagert und in äußeren Objekten gebunden werden. In diesem Sinne können wir die Natur bzw. die (DDR-)Gemeinschaft als positives Identifikationsobjekt, sozusagen als *Schiefheilungsschablone*, begreifen, das als gutes Objekt u. a. Wünsche nach Geborgenheit befriedigt, die von Cornelia selbst abgewehrt werden: Fürsorge verkommt in Cornelias Deutungssystem zur Irrationalität schlechthin, weil sie der Verweichlichung und Verängstigung der Kinder Vorschub leistet, wodurch sie keine angemessene Widerstandsfähigkeit entwickeln könnten: An ihren Nichten und Freundinnen kann sie aggressiv bearbeiten, was sie sich selbst nicht zugesteht. Zugleich werden leidvolle Erfahrungen und die damit einhergehenden Gefühlslagen wie bspw. die disziplinierenden Zugriffe ihres Vaters rationalisiert und mit Sinn erfüllt. Hinzu kommen gesellschaftliche Zwänge und krisenhafte biografische Erlebnisse, wie beispielsweise die Wende, die anhand der Schiefheilungsschablone mitverhandelt werden. Zentral ist hier eine doppelte Ohnmachtserfahrung, die sich über die gesellschaftlichen Umbruchserfahrungen der 1989/90er-Jahre einerseits (vgl. Haag und Hilmar, im Erscheinen) und deren Reaktivierung angesichts einer globalen Pandemie andererseits konstituiert. Diese doppelte Erfahrung der Ohnmacht verknüpft sich unter aktuellen Bedingungen mit Ängsten, welche um den eigenen sozialen Abstieg und den damit verbundenen Verlust eigner Freiheit und Lebensstandards zentriert sind (Amlinger und Nachtwey 2022; Forstenhäusler 2021, S. 55 f.). Neben den sozial bedingten Momenten kommt die Dynamik der Pandemie selbst als weiterer Faktor hinzu. So lässt sich etwa in der Pandemie das Natürliche, und damit auch das vermeintlich Rationale und ‚Gute‘, nicht mehr so eindeutig vom Unnatürlichen und Irrationalen trennen. Darüber hinaus wird die Natur selbst zur Bedrohung und droht als Quelle der Harmonie verloren zu gehen. In der conspirituality scheint sich das Verschwörungsdenken komplementärzu den esoterisch-spirituellen Vorstellungen zu verhalten: Das vermeintliche Gleichgewicht kann nur aus den Fugen geraten, weil außerordentlich mächtige Kräfte imaginiert werden, die am Werk sind, um einen ersehnten harmonischen Naturzustand sowie ein Gleichgewicht der drei Sphären aus den Angeln zu heben. Wenn auch personalisierende Zuschreibungen dieser Intentionen im Material weitgehend ausbleiben, ist das Interview doch über weite Strecke von einem verschwörungsideologischen Raunen durchzogen. Es lässt sich auf die Formel bringen, dass es Kräfte gebe, die daran arbeiten würden, die Menschen durch Propaganda zu verängstigen und zu lähmen, sie von einem „natürlichen Bezug zur Natur“ abzubringen und somit die ‚natürliche Ordnung‘ als solche zu stürzen. Auf dem WEF manifestiert sich diese Erosion insofern, als die Politik unzulässigerweise in das Schaffen der Wirtschaftstreibenden eingreift.

Dass die konstruierte Ordnung in der Krise an Stabilität eingebüßt hat, und dadurch neue Ängste geweckt oder vorhandene Ohnmachtserfahrungen bestärkt werden, befeuert das psychische und soziale Bedürfnis nach Halt nur noch zusätzlich. Damit aber die esoterisch-spirituelle Schiefheilungsschablone intakt bleibt, müssen Ängste und Verunsicherungen umso mehr projektiv abgesondert werden. In den von Cornelia beschworenen Szenarien ist es die korrumpierte Politik, die Ängste schürt, wogegen die in der Gestalt des Coronavirus auftretende Natur von jedweder Gefahr freigesprochen wird. Abgewehrt werden können dadurch ganz unterschiedliche Gefühle: Zum einen ist die vom Virus selbst ausgehende Gefahr gebannt, zum anderen stiften die Pandemie wie auch die Versuche, sie in den Griff zu bekommen, Unruhe und Unsicherheiten, die jedoch als ‚künstlich‘ den Verschwörern zugeschrieben werden können. Nur so lässt sich eine ambivalenzfreie (innere) Situation herstellen, in der man, wie Cornelia behauptet, von den Verheerungen der Pandemie, sowohl im Hinblick auf eigene Gesundheit als auch auf den eigenen sozialen Stand, weitgehend verschont bleiben konnte. Die Pandemie wird so in der Vorstellung Cornelias zu einem durch eine Verschwörung Erzeugtem und nicht zu einem Hereinbrechendem, dem sie ohnmächtig ausgeliefert wäre. Gerade die Betonung des Menschengemachten der Pandemie gibt ihr die Möglichkeit, eigene Ohnmachtserfahrungen abzuwehren und sich handlungsmächtig fühlen zu können.

Die Fiktion einer ‚Verschwörung gegen die Natur‘ bedient jedoch noch ein weiteres Bedürfnis: Im Angesicht dieser Bedrohungslage kann sich die Interviewte als rechtschaffene Widerstandskämpferin erleben. Dieser Widerstandsgestus lässt sich aus ihrer Modifizierung der Steiner’schen Lehren heraus begreifen. Das „Geistesleben“ wird im Zuge von Cornelias Aneignung der Dreigliederung durch das „Volk“ bzw. die harmonisch imaginierte Gemeinschaft ersetzt, die bei Steiner eigentlich in Differenz zum Geist steht. Bei Cornelia wird das bedrohte Volk, dessen Naturalisierung und Mythologisierung sich wiederum auch bei Steiner findet (Steiner 1976, S. 142, 1982b), zur Motivation des politischen Engagements. Obwohl Cornelia Verschwörungsideologien nicht laut propagiert, finden sich in ihrem Denken – durch die Verknüpfung von Natur und Volk, die psychologisch und biographisch emotional über die eigene Geschichte und die der Eltern aufgeladen ist, und die Idee einer diese natürliche Ordnung durcheinanderbringende Verschwörung – Momente des in der Corona-Protestszene weit verbreiteten ‚Great Reset‘-Verschwörungsnarrativs. Und obwohl sie sich in einer anderen Interview-Passage sehr explizit gegen ethnopluralistische Ideen richtet, tauchen in der Rede über die in den Westen „gelotsten“ Geflüchteten sogar Fragmente der rechtsextremen Verschwörungsideologie des ‚Great Replacements‘ auf, also der Phantasie, dass eine globale Elite über gelenkte Migration die Widerstandskraft von ‚Volksgemeinschaften‘ schwäche (Koblentz-Stenzler und Pack 2021). Gleichzeitig folgt aus dieser Aufspaltung von Natur und Verschwörung eine *Politikverschlossenheit*, da Politik in ihrer modernen Form als unnatürlich, nicht dem Volk dienend, wahrgenommen und den Verschwörern zugeschlagen wird. An der aktuellen Politik solle man demnach nicht partizipieren, sondern sich gegen ihre Zugriffe mit Entschlossenheit zur Wehr setzen.

## Fazit

Ziel dieses Artikels war es, das Verhältnis von Verschwörungsdenken und Esoterik bzw. Spiritualität einerseits in seinen historischen Wurzeln theoretisch darzustellen und andererseits anhand einer tiefenhermeneutischen Fallanalyse zu rekonstruieren, wie sich Elemente dieser Denkmuster bei Teilnehmenden der Corona-Proteste manifestieren können. Dabei wurde mit Hilfe des Konzepts der conspirituality in den Blick genommen, welche psychische Funktion die spezifische Verzahnung verschwörungsideologischer und esoterisch-spiritueller Denk- und Wahrnehmungsformen für die Protestierenden erfüllen kann.

Theoretisch konnten wir entwickeln, dass es sich bei der Verknüpfung von Verschwörungsdenken und Spiritualität keinesfalls um ein neues Phänomen handelt. Vielmehr greift die Interviewte Versatzstücke von Denkmustern auf, deren Wurzeln bis in die Aufklärung zurückreichen und die über die Zeit von subkulturellen Milieus ausgehend popularisiert wurden. Die Verbindung stellt sich somit vielmehr als eine dynamische dar, die immer neue historische Formen hervorbringt. Interessant ist hier, dass bei der Interviewten eine pessimistisch-apokalyptische Haltung überwiegt, im Gegensatz zu Elementen der New Age-Spiritualität, die eher von einem optimistischen Weltverhältnis geprägt sind und in der die sich anscheinend anbahnende Katastrophe als Chance einer neuen Weltordnung gesehen wird. Letztere sind bei ihr kaum ausgeprägt; für Cornelia stellt sich die aktuelle Situation vielmehr als fundamentale Bedrohung der ‚Natur‘ und der ‚natürlichen Ordnung‘ dar.

Dieses spezifische Verhältnis zu Natur und Natürlichkeit, das einen zentralen Bestandteil des Konzepts der conspirituality darstellt, blieb in aktuellen Analysen zum Thema weitgehend unterbeleuchtet (vgl. Schließler et al. 2020). Bei Cornelia präsentiert es sich jedoch als zentrales Element ihres Verhältnisses zur Welt: Die Natur wird in ihrer Erzählung sakralisiert; ihr wird eine harmonische Ordnung zugeschrieben, gegen die zu verstoßen tabuisiert wird, weshalb individuell ein ‚natürliches‘ Verhältnis zur Natur angestrebt wird, und ein solches auch als gesellschaftliches Leitbild ausgegeben wird. Dabei bedient sich Cornelia implizit des Modells der Dreigliederung des Sozialen Organismus von Steiner. Sie spricht von Volk, Politik und Wirtschaft als drei separaten Sphären, deren Vermischung den Kern der aktuellen Krise darstelle. In einer ‚natürlichen‘ Ordnung seien diese Sphären getrennt zu halten. Spirituell-esoterische Versatzstücke nimmt sie in ihre Beschreibung auf, um eine Problembeschreibung zu geben und zu bestimmen, welche Aspekte der ‚sakralen‘ Ordnung in der aktuellen Situation verletzt worden sind. Elementen des Verschwörungsdenkens bedient sich Cornelia, um zu artikulieren, welche Parteien sie in dieses ‚Sakrileg‘ verstrickt sieht.

Aus dieser historisch-theoretischen Einordnung ihrer Selbstdarstellung ergeben sich bereits erste Ansatzpunkte, die auf die sozial-psychologische Funktion der Verzahnung esoterisch-spiritueller und verschwörungsideologischer Denkmuster hinweisen, und die über den vorgestellten Einzelfall hinausweisen. So wurde bereits in der Vergangenheit Projektivität als zentrales Element der conspirituality identifiziert (vgl. Asprem und Dyrendal 2015; Schließler et al. 2020). Durch unsere Analyse konnten wir solche Bewegungen bei Cornelia als Schiefheilungsdynamiken beschreiben, die eine klare Trennung zwischen einer ‚guten Natur‘ und den die Ordnung bedrohenden Verschwörer:innen, und qua Identifikation damit auch zwischen dem ‚bösen Außen‘ und dem ‚guten Selbst‘ bzw. einer harmonisch imaginierten Gemeinschaft ermöglichen. Mit Bezug auf psychoanalytisch-sozialpsychologische Ansätze konnte gezeigt werden, dass der Reiz derartiger ‚Ordnungssysteme‘ darin liegt, dass mit ihrer Hilfe innere Konfliktlagen abgefedert werden können, indem Ängste, Ambivalenzen und Unsicherheit projektiv ausgestoßen werden. Als weiteres allgemeines Charakteristikum dieser Umgangsmuster konnten wir zeigen, dass in Krisenzeiten Schiefheilungstendenzen zusätzlich befeuert werden, einerseits weil die bislang stabilisierende Ordnung erschüttert wird, andererseits weil derartige Krisen akute Ängste, aber auch Aggressionen produzieren, mit denen ein Umgang gefunden werden muss. Darüber hinaus wird innerhalb der Protestszene Selbstaufwertung dadurch begünstigt, dass man sich als Trägerin heterodoxen Gegenwissens und im Widerstand gegen eine gegen die ‚natürliche Ordnung‘ verstoßende Elite wähnen kann.

Diese Überlegungen verdeutlichen Cornelias Politikverschlossenheit, vor allem gegenüber Politik in ihrer modernen Form. Während sie die DDR-Politik mit ihren scheinbar klaren Regeln und Vorgaben naturalisiert, geht die moderne Politik ihrer Ansicht nach ein unzulässiges Bündnis mit der Wirtschaft ein und überschreite somit ihre ‚natürliche‘ Grenze. Der Versuch, diese wahrgenommene Grenzüberschreitung zu verstehen, führt sie dazu, sich auch Momente von Verschwörungsideologien wie eines von ‚oben‘ orchestrierten ‚Great Resets‘ oder gar ‚Great Replacements‘ anzueignen. Das moderne kapitalistische Politiksystem ist in seinen Gesetzmäßigkeiten schwerer zu durchschauen, und die dafür charakteristische ‚Sprache der Macht‘ nicht mehr so leicht zu erlernen, wie in DDR-Zeiten. Psychodynamisch würde eine Vermischung der Sphären Politik und Wirtschaft von Cornelia Ambivalenztoleranz und Kompromissbereitschaft abverlangen; durch die projektive Trennung können negative Affekte ausgelagert werden und die Natur bzw. die Gemeinschaft als positives Identifizierungsobjekt erhalten bleiben. Um diese Gemeinschaft zu schützen, inszeniert sich Cornelia als Kämpferin für die Rechte der Schwachen, wodurch psychische Stabilisierung begünstigt wird.

Auf Grundlage unserer Analyse drängen sich folgende Facetten des Falles auf, die wir für die weitere Auseinandersetzung für zentral erachten: Bei der conspiritualistischen Vermengung verschwörungsideologischer und esoterisch-spiritueller Elemente handelt es sich keinesfalls um ein gänzlich neues Phänomen. Vielmehr werden Versatzstücke alter Denkmuster, die durchaus religiöse und spirituelle Züge aufweisen, auf neue Art kombiniert und an die aktuelle Situation angepasst. Zusammen mit der klaren Trennung von Gut und Böse, die sich aus den dargestellten psychischen Dynamiken ableitet, sorgt dies für psychische Entlastung, insbesondere in Krisenzeiten.

## Footnotes

1 New Age wird hier im weiteren Sinne als Sammelbegriff für Bewegungen verwendet, die sich seit den 1960er-Jahren formierten und Parallelen in Organisation und globaler Verbreitung aufweisen (Hanegraaff 1996; Knoblauch 2009). Dagegen fasst Hanegraaff unter New Age im engeren Sinne religiöse Bewegungen, die sich durch die „Erwartung eines epochalen Wandels zu einem ‚Neuen Zeitalter‘“ (Knoblauch 2009, S. 100) auszeichnen. Dies trifft nicht auf alle Bewegungen zu, die unter New Age im weiteren Sinne gefasst werden (Knoblauch 2009, S. 100 f.). Der Begriff ist trotzdem fruchtbar, da er erstens eine begriffliche Bestimmung der neueren religiösen Bewegungen seit den 1960ern und deren Gemeinsamkeiten erlaubt und zweitens den Akteur der von Knoblauch beschriebene Popularisierungsbewegung spiritueller Einstellungen benennen lässt.

2 Alle Transkriptausschnitte wurden nach dem Auswertungsprozess teilweise sprachlich geglättet, um den Lesefluss nicht zu stören.

3 Mit einer Unterbrechung nach Steiners Tod zeigt sich seit den 1960er-Jahren eine politische, z. T. selektive Rezeption von Steiners Schriften zur sozialen Dreigliederung in verschiedenen Konstellationen, politischen Bündnissen und Parteien, die sich für direkte Demokratie und das bedingungsloseGrundeinkommen einsetzen (Zander 2019, S. 175 ff.). Auch die Steiners Anthroposophie zeigt eine unter dem Begriff der populären Spiritualität zu fassende Verselbständigung und Dissemination der Konzepte über das organisierte anthroposophische Milieu hinaus. Grob lässt sich die Rezeption nach Zander in vier Dimensionen unterteilen: Eine *libertäre*, eine rechts-konservative, eine *säkularisierende* und eine *esoterische *(ebd., S. 181 ff). Diese Dimensionen sind, wie Martins feststellt, bereits im abstrakten Konzept der Dreigliederung angelegt, welchem Steiner in seinen Vorträgen je nach Publikum eine autoritäre oder liberale Schlagrichtung gegeben hat (Martins 2012, S. 122). In den Waldorfschulen, anderen Institutionen und Initiativen existiert die die Dreigliederung zudem in der Praxis fort. Zeitgleich hat die Dreigliederung als politisches Konzept in dezidiert anthroposophischen Kreisen zugunsten der esoterischen Lehre Steiners weitestgehend an Bedeutung verloren (Zander 2019, S. 185 f.).

4 Bemerkenswerterweise verweist auch die Interviewte auf die besondere Rolle der Bildung in ihrer Jugend. So spricht sie unter anderem von einem „phantastischen Bildungssystem“ in der DDR, in der die Grundlagen für den Erwerb der besagten „Fähigkeiten und Fertigkeiten“ gelegt worden seien.
